# Delayed Femoral Nerve Palsy Associated with Iliopsoas Hematoma after Primary Total Hip Arthroplasty

**DOI:** 10.1155/2016/6963542

**Published:** 2016-09-26

**Authors:** Sandeep Kumar, Gerald Pflueger

**Affiliations:** ^1^Department of Orthopaedics, Evangelisches Hospital, Vienna, Austria; ^2^University of Vienna, Vienna, Austria; ^3^Evangelisches Hospital, Vienna, Austria

## Abstract

Femoral nerve neuropathy after total hip arthroplasty is rare but catastrophic complication. Pain and quadriceps muscle weakness caused by this complication can significantly affect the functional outcome. Here we present a case report, describing delayed onset femoral nerve palsy associated with iliopsoas hematoma following pseudoaneurysm of a branch of profunda femoris artery after 3 months of primary total hip arthroplasty in an 80-year-old female patient with single kidney. Hip arthroplasty was done for painful primary osteoarthritis of left hip. Diagnosis of femoral nerve palsy was made by clinical examination and computed tomography imaging of pelvis. Patient was managed by surgical evacuation of hematoma and physiotherapy. The patient's clinical symptoms were improved after surgical evacuation of hematoma. This is the first case report of its kind in English literature regarding delayed onset femoral nerve palsy after primary total hip arthroplasty due to pseudoaneurysm of a branch of profunda femoris artery without any obvious precipitating factor.

## 1. Introduction

Femoral nerve neuropathy after total hip arthroplasty is rare but catastrophic complication. The incidence rate ranges from 0.1 to 2.4% in primary hip arthroplasty [1]. Pain and quadriceps muscle weakness caused by this complication can significantly affect the functional outcome.

Possible etiology for femoral nerve palsy reported in the literature are drilling through the medial cortex of acetabulum, cement extrusion, hardware irritation like screws, postoperative use of anticoagulation agents, and coagulation abnormality like hemophilia.

This case report describes a delayed onset iliopsoas hematoma development due to pseudoaneurysm of a branch of profunda femoris artery, which results in femoral nerve palsy in uncemented primary hip arthroplasty patient. This is the first case report of its kind in English literature regarding delayed onset femoral nerve palsy after total hip arthroplasty due to pseudoaneurysm of a branch of profunda femoris artery without any obvious precipitating factor. We have described the clinical evaluation, investigation, and management of delayed onset femoral nerve palsy after total hip arthroplasty in an old age patient with single kidney.

## 2. Case Report

**Figure 1:**
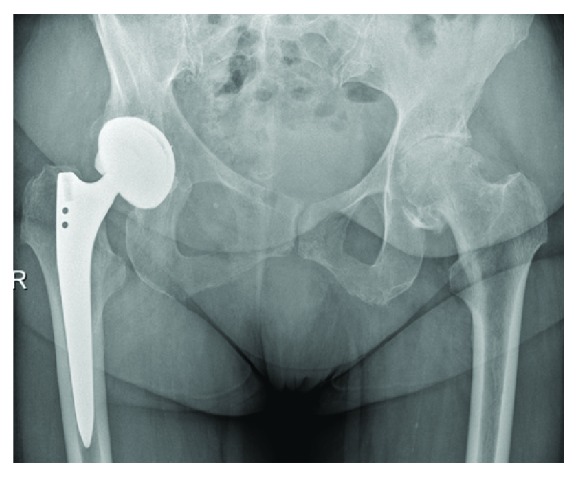
Preoperative X-ray showing the osteoarthritis of left hip.

An 80-year-old female patient underwent total hip replacement for primary osteoarthritis of left hip through minimally invasive Watson jones approach (Figure 1). During the surgery uncemented press fit acetabular cup and uncemented femoral stem were used. We did not use any screw for acetabular cup fixation. Immediate postoperative period was uneventful and patient was discharged from the hospital 6 days after surgery. As per our hospital protocol we use low molecular weight heparin for 6 weeks for thromboprophylaxis in total hip replacement patients.

After 3 months of surgery, patient came back with the complaint of pain in the left inguinal region and development of ecchymosis over the left inguinal region. Patient was readmitted to the hospital for observation and further investigation. Next morning patient started complaining of weakness of left lower limb and numbness over medial aspect of thigh and knee. On physical examination, the patient was found to have grade 3/5 motor function of the quadriceps muscle, as well as decreased light touch and pinprick sensation over the femoral nerve distribution area. Patient did not have any spine tenderness.

**Figure 2:**
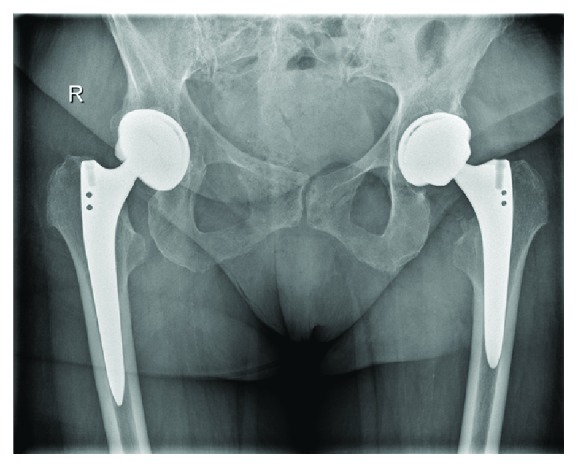
Postoperative X-ray showing left total hip arthroplasty.

**Figure 3:**
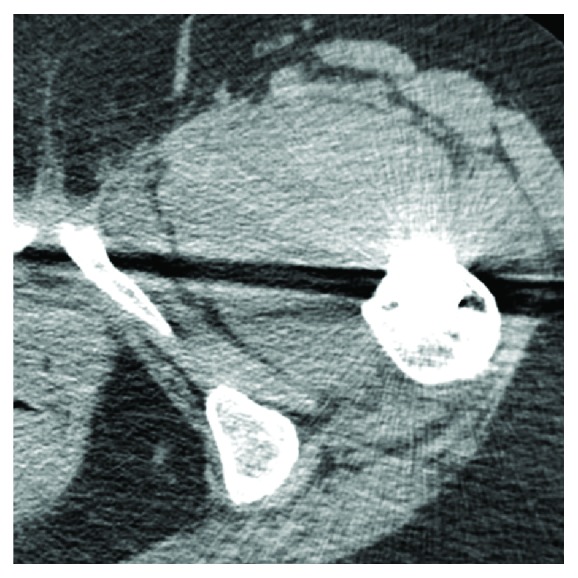
Axial section of CT scan showing hematoma in iliopsoas region.

**Figure 4:**
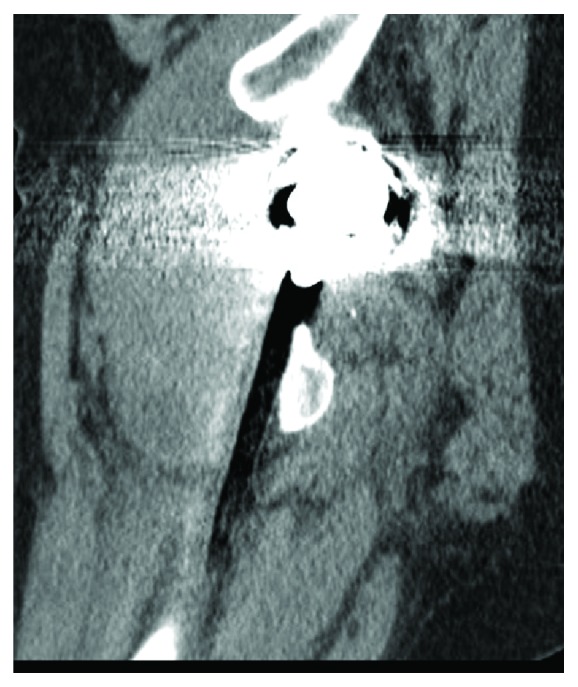
Sagittal section of CT scan showing the hematoma.

Blood investigations showed mild anemia with haemoglobin level of 9.0 g/dL. There were no abnormal findings of bleeding tendency or disorders of hemostasis and she had not been treated with any anticoagulant drugs. The anteroposterior and lateral radiographs of the pelvis and left hip showed no abnormality except previously performed total hip replacement, with no displacement or dislocation of the prosthesis noted (Figure 2). A radiograph of lower spine was normal. MRI of lower spine was normal. Computerized tomography (CT) examination of pelvis showed 8 × 10 cm mass around the left lesser trochanter extending in thigh in vastus intermedius muscle (Figures 3 and 4). So the case was diagnosed as femoral nerve palsy due to delayed hematoma development in iliopsoas region. We have tried to aspirate the hematoma under sterile precautions but only 6 mL of occult blood comes out. CT guided aspiration of hematoma was refused by radiologist as hematoma was organized. Surgical decompression was planned after opinion of vascular surgeon.

Incision was given through direct anterior approach. A hematoma was found in the lesser trochanter region and in front of joint. The femoral nerve was found stretched by the hematoma. Femoral nerve and artery were identified and secured with mersilene tape. The organized hematoma around 150 gms was removed. Fresh bleeding starts once the hematoma was removed, because the ruptured pseudoaneurysm was occluded only by tamponade effect of the hematoma. Bleeding vessel was identified as branch of profunda femoris artery and sutured. A drain was kept in situ and closure was done. Postoperative culture reports of hematoma were sterile. The inguinal pain was relieved completely after the surgery. Patient was started walking with knee brace. The power of the quadriceps femoris recovered gradually.

## 3. Discussion

Femoral nerve palsy after iliacus hematoma is common in hemophilic patients [2]. But femoral nerve palsy after total hip replacement is a rare complication. Most of the time it is an intraoperative complication. Schmalzried et al. [3] noted that the exact cause of nerve injury is rarely identified with absolute certainty. In a study by Farrell et al. [4], only 55% of the patients had a presumably identifiable cause of the nerve injury. Previously reported causes of femoral nerve palsy after total hip replacement could include over lengthening [5], compression from a hematoma [6], from extruded cement [7], and laceration from a screw used in the acetabular component [8].

Incidence rate of femoral nerve palsy is higher in revision hip replacement surgeries as compared to primary total hip replacement surgery [1]. Delayed femoral nerve palsy after primary total hip replacement is very rare complication. In our case femoral nerve palsy occurs following hematoma development in iliopsoas region around lesser trochanter after three months of primary hip replacement. We believe that application of retractors around lesser trochanter during the surgery causes minimal damage to the arterial wall of a branch of profunda femoris artery that leads to pseudoaneurysm development and delayed hemorrhage later on causes femoral nerve palsy.

Nakamura et al. [9] reported a case of femoral nerve palsy associated with iliacus hematoma following pseudoaneurysm after revision hip arthroplasty. They emphasized the use of CT angiography for the detection of pseudoaneurysm and active bleeding; we did not use that modality because our patient has single kidney and contrast agent can be harmful. The noncontrast CT scan gave us sufficient information about hematoma.

Because of rareness of the condition, treatment of iliopsoas hematoma with associated femoral neuropathy after total hip arthroplasty remains controversial. In hemophilia cases, conservative therapy associated with favorable outcome [2]. Gogus et al. [6] reported good results with nonsurgical treatment of iliacus hematoma following primary hip arthroplasty. The other alternative option is percutaneous aspiration but that is not useful for organized hematomas. We believe that, for patient with delayed femoral nerve palsy following total hip arthroplasty, early operative intervention should be undertaken specially in cases without disorder of haemostasis.

We emphasizes the judicious use of retractors during surgery, careful clinical examination in postoperative period if patient complains of inguinal pain and radiating pain to thigh, and early use of radiological investigation like CT scan for prompt diagnosis. We also advocate the early surgical intervention and evacuation of hematoma to prevent the further damage of nerve and to relieve pain caused by stretching of nerve.
